# Seaweed Derived Lipids Are a Potential Anti-Inflammatory Agent: A Review

**DOI:** 10.3390/ijerph20010730

**Published:** 2022-12-30

**Authors:** Agnieszka Jaworowska, Aliza Murtaza

**Affiliations:** 1 Division of Medicine, University College London, London WC1E 6BT, UK; 2 School of Science, University of Greenwich, Chatham ME4 4TG, UK

**Keywords:** anti-inflammatory, seaweeds, macroalgae, inflammation, lipids, fatty acids, omega 3

## Abstract

Chronic, low-grade inflammation is linked to the development of non-communicable diseases, including cancer, cardiovascular disease, obesity, insulin resistance, diabetes, and others which together contribute to more than 50% of deaths globally. Modulation of inflammatory responses may be a promising strategy, and n-3 long chain polyunsaturated fatty acids (n-3 LC-PUFA) may offer a new therapeutic option in inflammatory conditions. Seaweeds are characterised by high nutritional quality and are a good source of many bioactive compounds, including n-3 LC-PUFA. This review addresses the potential anti-inflammatory properties of seaweed derived lipids, and their immunomodulating mechanisms in order to identify the possible applications of seaweed as an anti-inflammatory functional food ingredient or dietary supplement. A few studies have evaluated the anti-inflammatory activity of seaweed lipids using crude lipid extracts, lipid fractions and isolated complex lipids from several seaweeds belonging to the Ochrophyta and Rhodophyta phyla, with only three *Ulva rigida*, *Ulva* sp. and *Codium tomentosum* within the Chlorophyta phylum. It was reported that seaweed derived lipids suppress inducible nitric oxide synthase and cyclooxygenase-2 expression and reduce nuclear factor κB p100 and myeloid differentiation primary response 88 protein levels leading to the downregulation of the production of several pro-inflammatory cytokines and nitric oxide. Further investigations are required to unravel the complex mechanisms underlying their preventive action against chronic inflammation and their potential use as a new functional food ingredient and/or health supplement.

## 1. Introduction

Inflammation is the natural protective response of the immune system of living organisms to infection and other pathogenic factors. It involves a cascade of cellular and microvascular reactions to eliminate pathogens and stimulate tissue repair processes resulting in the restoration of homeostasis at infected and/or injured sites [1,2,3]. The inflammatory response is initiated by an interaction between pattern recognition receptors (PRRs) and conserved molecular structures of pathogens (pathogen-associated molecular patterns (PAMPs)) or damage-associated molecular patterns (DAMPs) [4]. PRRs activation initiates an intracellular signaling cascade resulting in the nuclear translocation of various transcription factors, including activator protein-1 (AP-1) and nuclear factor κB (NF-κB) or interferon regulatory factor 3 (IRF3) [5]. It triggers the production of a number of inflammatory mediators such as lipid mediators (prostaglandins, leukotrienes, and platelet-activating factor), inflammatory cytokines (tumor necrosis factor (TNF)-α, interleukin (IL)-1β, IL-6), vasoactive amines and peptides (histamine, serotonin), acute phase proteins (C-reactive protein (CRP), serum amyloid A (SAA), complement proteins), reactive oxygen spices and enzymes that in turn alter the functionality of the target tissues and organs [6].

Normally, inflammation is beneficial to the host and the inflammatory response is self-limiting resolving rapidly when the triggering insult has been eliminated, the infection is cleared, and damaged tissue is repaired. However, if the inflammatory response becomes dysregulated the activated immune system can cause irreparable damage to the host tissues and organs resulting in adverse health outcomes [7]. Many lines of evidence indicate that various health problems may arise from chronic, dysregulated inflammatory responses, including multiple sclerosis, cancer, rheumatoid arthritis, atherosclerosis, cardiovascular disease, obesity, dermatitis, migraine, irritable bowel disease, insulin resistance, diabetes, and others [8,9,10]. Clinical and epidemiological studies have reported that elevated levels of IL-6, CRP, IL-1β are predictive of the development of type 2 diabetes and cardiovascular events [11,12,13,14]. These chronic, non-communicable diseases (NCDs) are the main cause of public health challenges around the world resulting in illness, economic loss, and poor quality of life [15,16]. The WHO predicts that over 70% of all deaths globally will be related to NCDs by 2025, and an estimated 41 million people will die from NCDs, including cardiovascular disease ((CVDs), (48%)), cancers (21%), chronic respiratory disease (12%) and diabetes (3%) [17]. The evidence indicates that targeting inflammatory pathways could provide a new opportunity to treat and/or prevent chronic metabolic disorders and warrants further investigations in order to develop novel and effective anti-inflammatory agents.

The application of drugs targeting inflammatory pathways appears to be a promising strategy in type 2 diabetes and CVD treatment [18,19]. However, long-duration pharmacological anti-inflammatory treatment may not be an ideal therapy in terms of adverse effects on the host defense system and long-term health outcomes [20,21,22]. Alternatively, several nutrients and bioactive compounds present in the diet have shown significant anti-inflammatory activity and may offer a safer option than pharmaceutical treatment, especially for long-term use. [23,24]. One of the most extensively studied nutrients, in relation to their immunomodulating properties, are dietary fatty acids. Long chain polyunsaturated n-3 fatty acids (LC-PUFA), including eicosapentaenoic acid (EPA) and docosahexaenoic acid (DHA) exhibit anti-inflammatory activities [25], while some saturated fatty acids activate Toll-like receptor 4 (TLR4) and trigger inflammatory response [26,27]. EPA and DHA bind to G protein-coupled receptor (GPR120) [28] to inhibit NF-κB activation and up-regulate peroxisome proliferator activated receptor (PPAR)-γ, resulting in reduced secretion of pro-inflammatory cytokines [29]. Furthermore, marine-derived n-3 LC-PUFAs supplementation reduces blood concentrations of CRP, IL-6, and TNF-α [30].

Taking into account anti-inflammatory effects, n-3 LC-PUFAs may offer an important therapeutic option in inflammatory diseases. EPA and DHA can be derived from alpha-linolenic acid (ALA) through multiple enzymatic elongation and desaturation reactions catalysed by delta-5 and delta-6 desaturases. However, the conversion rate of ALA to DHA in humans is very low and depends on a number of external and internal factors [31,32]. Therefore, in humans DHA should be obtained from the diet by eating fish and/or other types of seafood, and plasma concentration of DHA is lower in vegetarians and vegans than in omnivores [33].

The global supply of n-3 LC-PUFAs from all known traditional sources, including capture fisheries and aquaculture, is insufficient to meet human nutritional requirements [34]. Thus, alternative new food sources of EPA and DHA are needed, and seaweeds may present an innovative source of these anti-inflammatory fatty acids [35,36,37]. Seaweeds have been used as food and in traditional medicine since ancient times in Asian countries [38] and in the last decade interest in the cultivation and use of seaweeds for human nutrition and health has grown globally [39,40,41]. Seaweeds are characterized by high nutritional quality [40,42] and are a good source of many bioactive compounds [43] with antibacterial, antiviral, antifungal, anti-oxidant, antitumor and anti-inflammatory activities [44,45]. They are also a well-known reservoir of polyunsaturated fatty acids, including EPA and DHA [39,40,46].

This review addresses the potential anti-inflammatory properties of crude lipid extracts, lipid fractions, and isolated lipids from seaweeds and their immunomodulating mechanisms in order to identify the possible application of seaweed as an anti-inflammatory functional food ingredient or dietary supplement. In addition, this review provides a comprehensive overview of fatty acids composition, including the n-6:n-3 ratio, of different seaweed species to assess their usefulness as a dietary source of polyunsaturated fatty acids.

## 2. Methods

**Figure 1:**
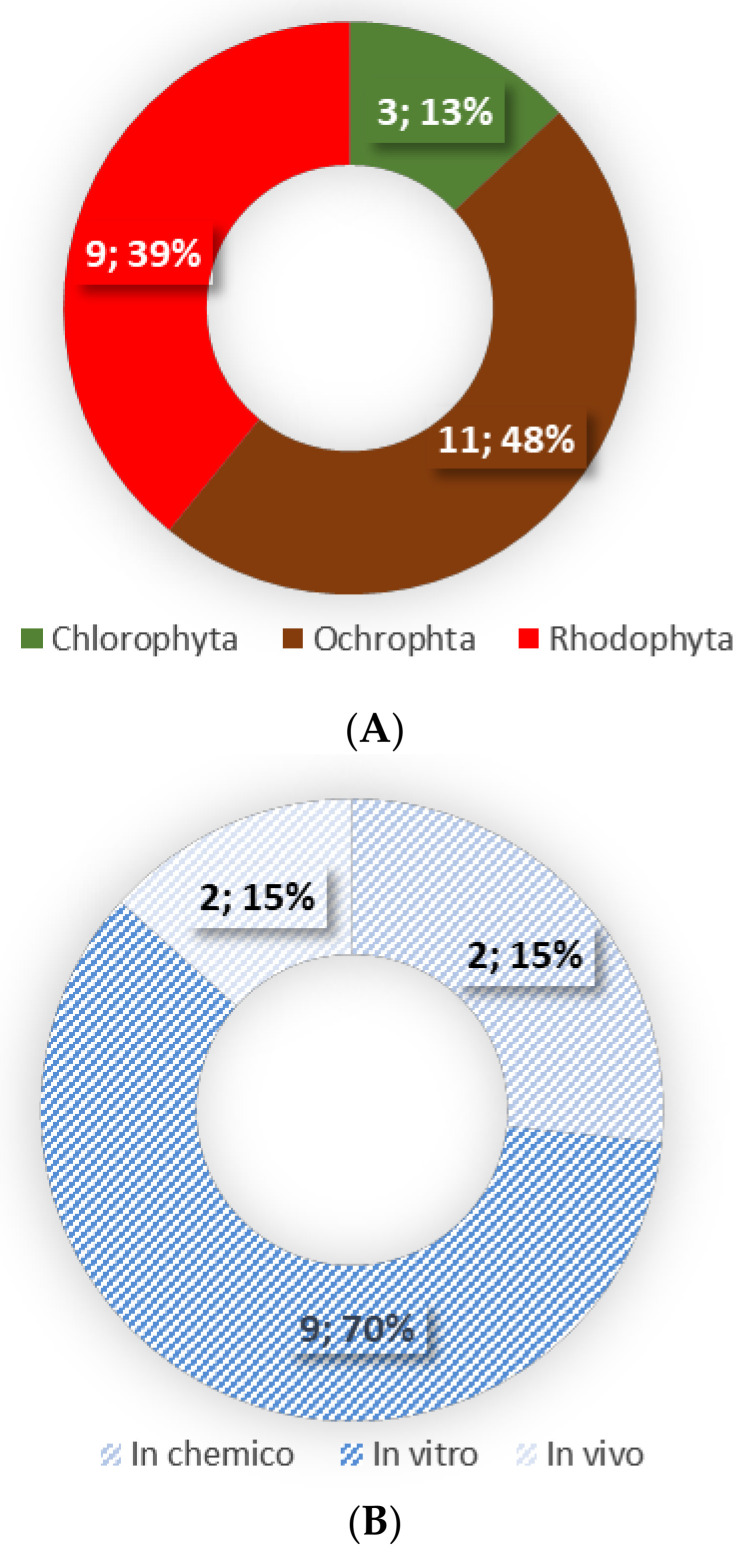
(**A**) Seaweed phyla (expressed as number; % of all studied seaweeds) and (**B**) type of performed assays (expressed as number; % of research articles) used to evaluate the anti-inflammatory activity of seaweed lipids.

This review is based on the literature search performed in the scientific databases of PubMed, Web of Science, Scopus and Science Direct, using the combination of the following keywords: (1) macroalgae or seaweed or algae and (2) lipids or lipid extracts or complex lipids or fatty acids and (3) anti-inflammatory or immunomodulatory. Only original research articles using lipid extracts, lipid fractions and/or isolated lipids of seaweeds were included in this review. In total 13 publications were included (Figure 1).

## 3. Definition and Classification of Seaweeds

Seaweeds are marine photosynthetic macroalgae that grow in various types of water and can be found in any climatic zone. They can be attached to rocks, pebbles, and other substrata or freely floating primitive plants that lack true root, stem, and leaves [46]. Seaweeds are classified into three groups based on their pigment composition: Ochrophyta (class Phaeophyceae, brown algae), Rhodophyta (red algae) and Chlorophyta (green algae) [47], with more than 10,000 different seaweed species identified worldwide [48]. The colour of brown algae varies from brown to yellow depending on the content of fucoxanthin that masks chlorophyll a and b, and other xanthophylls. Red algae colour varies from red to reddish-brown and purple due to the dominance of phycobiliproteins, especially phycoerythrin (red) and phycocyanin (blue-green), and in the case of green algae, their green colour is due to the presence of chlorophyll a and b [49,50]. Red seaweeds are the largest group of algae with 6100 known species and are efficient in photosynthesizing in deeper waters. On the other hand, green seaweeds (2200 known species) are common in areas where light is abundant, and the main genera includes, *Ulva*, *Codium*, *Chaetomorpha*, and *Cladophora*. There are around 1800 species of brown seaweeds, with two main orders *Fucales* and *Laminariales*, and less than 1% are found in freshwater [39,51].

## 4. Seaweeds in Human Diet

Seaweeds have been harvested and used as food since ancient times in Asian countries, such as Korea, China, and especially Japan [38]. There are over 600 recognized edible seaweed species [52] and around 200 are consumed worldwide, with brown seaweeds being the most common edible seaweed (66.5%), followed by red (33%) and green (5%) [48,53]. The most popular seaweed species are nori (*Porphyra*/*Pyropia* sp.), wakame (*Undaria* sp.), kombu (*Saccharina*/*Laminaria* sp.), and dulse (*Palmaria palmata*) [40,54,55]. It is estimated that approximately one-fifth of meals consumed in Japan contain seaweed [40]. In Western countries, traditionally, seaweed intake has been limited to coastal communities, but in recent years interest in the cultivation and use of seaweeds, especially as nutraceuticals and functional food, in the West has grown [39,40,41]. In fact, the application of seaweeds to develop new food products increased by 147% between 2011 and 2015 in the European market [39,56]. Edible seaweeds are commercially available in a variety of food products, including both whole seaweed (fresh and dry) or seaweeds added as a functional ingredient to bread, confectionery, condiments, drinks, noodles and pasta, salads, snacks, soup, supplements, sushi, dairy, fish and meat products [39,42].

Seaweeds are characterized by high nutritional quality and are a good dietary source of fat- and water-soluble vitamins and essential minerals (calcium, iron, iodine, magnesium, phosphorous, potassium, zinc, copper, manganese, selenium and fluoride), with some seaweeds containing 10–100 more minerals and vitamins than terrestrial plants or animal food products [40,42,57,58].

They are also a good source of proteins (5–47% of dry seaweed mass, with the highest content in red seaweeds and the lowest in brown), essential amino acids and fibre (36–60% of dry seaweed mass). Lipid content of seaweeds is low, but rich in EPA and DHA [39,40,46]. This nutritional characteristic makes seaweed a healthy, nutrient-dense, and low energy food and an attractive ingredient for functional food use. In addition, seaweeds are a good source of several non-nutrient bioactive compounds such as polyphenols, sterols, flavonoids, tannins, pigments, polysaccharides, and terpenes [59]. It has been reported that these biologically active components may help to treat and/or prevent public health problems, due to their antibacterial, antiviral, antifungal, antioxidant, anti-inflammatory and antitumor activities [59,60,61]. Indeed, studies have shown that regular consumption of seaweeds was inversely associated with ischemic heart disease, mortality from stroke, and several cancers [62,63,64]. Historically, seaweeds have been used in traditional medicine, especially in East Asian countries [65], with 171 species of medicinal algae documented [58]. Some of the species of Sargassum have been successfully used to treat the symptoms of inflammation associated disorders such as the painful scrotum, edema, liver organ disease and chronic bronchitis [66,67]. According to the Korean Medical Textbook, seaweeds have been used to treat edema and painful scrotum [66].

## 5. Seaweed Lipids and Fatty Acids Composition

Lipid content in macroalgae is considered low and ranges from 0.5 to 8.0% of dry weight [46,68], with neutral lipids (fatty acids, triglycerides, and sterols), glycolipids (GL) and phospholipids (PL) being the main lipid classes [39,69,70]. Seaweed lipids concentration and composition varies significantly between and within species, and depends on geographical region, season, and environmental conditions, with temperature being the major factor [71,72,73].

Despite the low-fat content, seaweeds are recognised as a rich source of biologically active lipids with a high proportion of unsaturated fatty acids, including n-3 LC-PUFA. In seaweeds, fatty acids are mainly found as polar lipids, including GL, PL, and betaine lipids [68]. The fatty acids composition of different seaweeds expressed as % of total fatty acids of the seaweed total lipid extracts is shown in Table 1. The proportion of saturated fatty acids (SFAs) varies widely from 7.53 to 95.21%, monounsaturated fatty acids (MUFAs) from 2.30 to 47.10%, and PUFAs from 2.60 to 73.70% (Table 1). Red seaweeds are characterized by the highest content of SFAs (46.90 ± 15.73)% followed by green (45.33 ± 15.96)% and brown algae (38.00 ± 13.31)%. On the other hand, the phylum Ochrophyta is the richest source of PUFAs (34.59 ± 15.58)%, with slightly lower content in the Rhodophyta (28.34 ± 16.26)% and the Chlorophyta (27.26 ± 14.22)%. Palmitic acid is the dominant fatty acid of seaweed, with its content ranging from 4.24 to 84.60% of the total FA fraction, followed by oleic acid 0.15–46.00% across all phyla (Table 1). Seaweeds are also an important source of the essential PUFAs such as alpha-linolenic acid (ALA, C18:3, n-3) and linoleic acid (AL, C18:2, n-6), which cannot be synthesized by mammals, with a range between 0.10 and 15.20%, and between 0.16 and 28.60% of total FA content, respectively. In general, green macroalgae had higher ALA and AL content (5.06 ± 4.90% and 7.40 ± 6.04%, respectively) than brown (4.41 ± 2.70% and 5.97 ± 3.47%) and red algae (1.93 ± 3.26% and 1.93 ± 1.23%). Macroalgae can also synthesise LC-PUFAs such as n-6 AA and n-3 EPA and DHA. It is worth noting that DHA and EPA are produced in significant quantities only in the marine environment, including macroalgae. Endogenous synthesis in humans is limited and may not meet the metabolic demand [74]. The main n-3 LC-PUFA in many macroalgae is EPA representing 21.17 ± 15.71% of total FA content in Rhodophyta 7.81 ± 6.92% in Ochrophyta and 1.82 ± 1.56% in Chlorophyta. On average, the DHA content in macroalgae is lower than EPA and ranges between 6.91 ± 16.76% in Rhodophyta, 3.56 ± 7.77% in Chlorophyta and 2.16 ± 4.19% in Ochrophyta. The highest concentration of DHA was reported in the red algae *Gracilaria changii* (48.36 ± 6.76)%. Both n-6 and n-3 PUFAs are essential for human health and the recommended ratio between n-6:n-3 is below 1:1–4:1 to reduce the risk of cardiovascular, inflammatory and neurological conditions [75]. The average n-6:n-3 ratio is the lowest in the green species (1.02 ± 1.32) followed by brown (1.29 ± 0.83), and red macroalgae (1.84 ± 3.36) and is within the recommended n-6:n-3 ratio to prevent the chronic diseases [75].

In addition, seaweeds are also the source of other bioactive lipids including squalene and phytosterols such as brassicasterol, chondrillasterol, fucosterol and isofucosterol [69,76] with reported antioxidant, anti-cancer, and anti-inflammatory activities [77].

**Table 1:** Fatty acid composition expressed as % of total fatty acids (mean ± standard deviation (SD)) of different seaweed species.

	SFA	C16:0	C18:0	MUFA	C16:1	C18:1	PUFA	C18:2 (LA)	C18:3 (ALA)	C20:4 (ARA)	C20:5 (EPA)	C22:6 (DHA)	n-6/ n-3
CHLOROPHYTA	46.87 ± 17.78	31.17 ± 11.55	5.90 ± 9.68	16.90 ± 8.05	4.02 ± 3.26	10.24 ± 5.93	27.56 ± 15.05	7.92 ± 6.65	5.26 ± 4.91	2.16 ± 2.35	1.82 ± 1.56	3.56 ± 7.76	1.02 ± 1.32
Caulerpaceae	49.26 ± 22.51	33.67 ± 10.18	5.25 ± 5.70	19.67 ± 13.45	5.72 ± 2.17	17.35 ± 9.42	18.86 ± 7.54	5.99 ± 2.80	7.50 ± 2.47	1.49 ± 1.17	1.59 ± 0.90	n.d.a	1.14 ± 0.89
*Caulerpa lentillifera* [78]	29.80 ± 1.65	33.69 ± 0.64	13.57 ± 0.91	9.08 ± 2.75	n.d.a	n.d.a	13.06 ± 0.32	n.d.a	n.d.a	2.84 ± 0.53	n.d.a	n.d.a	0.79 ± 0.05
*Caulerpa racemosa* [79,80]	64.68 ± 33.07	39.16 ± 16.35	3.40 ± 0.14	18.45 ± 22.82	3.97 ± 0.74	n.d.a	15.61 ± 8.47	6.34 ± 3.75	4.89 ± 3.25	n.d.a	n.d.a	n.d.a	1.84 ± 1.23
Cladophoraceae	47.90 ± 10.79	28.49 ± 6.32	0.82 ± 0.40	21.49 ± 6.78	5.62 ± 5.07	12.55 ± 4.02	23.77 ± 8.90	17.69 ± 11.74	5.20 ± 4.84	0.89 ± 0.56	n.d.a	n.d.a	n.d.a
*Chaetomorpha linum* [37,81]	n.d.a	27.95 ± 7.00	n.d.a	n.d.a	2.50 ± 2.54	13.05 ± 6.15	n.d.a	15.35 ± 18.73	2.55 ± 2.19	0.65 ± 0.77	0.95 ± 0.49	1.15 ± 1.06	2.73 ± 1.37
*Cladophora albida* [82]	50.03 ± 0.56	33.04 ± 0.52	1.28 ± 0.19	27.73 ± 0.11	13.90 ± 0.09	12.51 ± 0.02	22.24 ± 0.24	15.54 ± 0.22	n.d.a	1.37 ± 0.07	2.02 ± 0.05	0.86 ± 0.03	6.73
*Cladophora glomerata* [36]	14.10 ± 2.31	44.28 ± 0.81	0.92 ± 0.05	62.52 ± 11.48	0.37 ± 0.02	20.71 ± 1.65	23.37 ± 4.60	8.19 ± 0.82	1.12 ± 0.12	1.43 ± 0.04	n.d.a	n.d.a	n.d.a
*Cladophora Rupestris* [83]	40.80 ± 0.50	31.80 ± 2.80	n.d.a	27.30 ± 1.00	8.30 ± 1.10	10.50 ± 2.10	21.50 ± 3.10	7.40 ± 0.80	5.20 ± 1.70	n.d.a	2.50 ± 0.60	n.d.a	0.50 ± 0.00
*Rhizoclonium riparium* [37]	34.40 ± 0.10	20.30 ± 0.20	0.40 ± 0.00	23.00 ± 0.2	6.40 ± 0.00	15.70 ± 0.20	33.10 ± 0.40	n.d.a	10.50 ± 0.00	0.90 ± 0.00	2.70 ± 0.00	0.40 ± 0.00	1.70 ± 0.10
Codiaceae	46.28 ± 22.74	30.35 ± 9.43	1.87 ± 0.63	16.04 ± 1.06	5.55 ± 0.20	8.75 ± 6.41	37.67 ± 21.67	6.19 ± 3.49	2.40 ± 0.42	3.95 ± 0.77	4.69 ± 4.53	n.d.a	1.12 ± 1.27
*Codium fragile* [82]	62.37 ± 1.50	40.73 ± 0.83	1.51 ± 0.06	15.29 ± 0.97	5.41 ± 0.17	0.40 ± 0.94	22.34 ± 0.55	9.21 ± 0.32	n.d.a	3.41 ± 0.20	1.48 ± 0.17	n.d.a	2.02
*Codium isthmocladum* [84]	n.d.a	28.03 ± 2.67	1.50 ± 0.12	n.d.a	n.d.a	13.66 ± 1.35	n.d.a	2.36 ± 0.18	2.10 ± 0.19	n.d.a	n.d.a	n.d.a	n.d.a
*Codium tomentosum* [85]	30.20 ± 1.60	22.30 ± 1.20	2.60 ± 0.60	16.80 ± 0.30	4.90 ± 0.20	11.10 ± 0.40	53.00 ± 1.40	3.40 ± 0.10	14.00 ± 0.60	n.d.a	7.90 ± 0.80	n.d.a	0.22
Ulvaceae	46.90 ± 19.28	34.26 ± 12.36	7.98 ± 12.18	14.91 ± 6.93	3.22 ± 2.86	9.85 ± 4.59	28.15 ± 16.35	7.28 ± 4.83	5.65 ± 5.62	2.50 ± 2.79	1.41 ± 0.82	1.37 ± 1.34	0.62 ± 0.40
*Enteromorpha compressa* [81]	n.d.a	23.10	n.d.a	n.d.a	1.10	6.30	53.90	3.80	21.90	0.50	1.40	n.d.a	n.d.a
*Enteromorpha intestinalis* [35,83]	42.80 ± 25.17	31.05 ± 11.10	n.d.a	23.60 ± 1.69	4.85 ± 4.31	12.35 ± 4.03	25.95 ± 15.76	7.10 ± 1.83	7.85 ± 9.26	n.d.a	0.55 ± 0.35	n.d.a	0.35 ± 0.21
*Ulva prolifera* [37]	38.10 ± 0.20	21.00 ± 0.20	0.20 ± 0.00	14.90 ± 0.10	1.60 ± 0.10	11.50 ± 0.20	39.00 ± 0.5	22.00 ± 0.80	0.20 ± 0.10	1.70 ± 0.00	2.20 ± 0.10	0.20 ± 0.00	0.60 ± 0.00
*Ulva armoricana* [69]	46.50 ± 0.10	42.00 ± 0.20	1.00 ± 0.10	24.30 ± 0.10	n.d.a	17.30 ± 0.10	29.20 ± 0.10	8.40 ± 0.10	0.50 ± 0.10	Trace	Trace	n.d.a	0.10 ± 0.10
*Ulva Australia* [78]	25.67 ± 0.47	24.37 ± 0.35	35.41 ± 0.44	3.45 ± 0.24	n.d.a	n.d.a	36.23 ± 0.82	n.d.a	n.d.a	4.23 ± 0.08	n.d.a	n.d.a	0.25 ± 0.01
*Ulva chaugulii* [86]	51.40 ± 0.11	44.60 ± 0.20	1.10 ± 0.05	19.40 ± 0.10	6.40 ± 0.03	8.90 ± 0.03	29.20 ± 0.10	7.00 ± 0.10	11.00 ± 0.04	0.70 ± 0.01	1.20 ± 0.01	1.10 ± 0.10	0.50 ± 0.00
*Ulva fasciata* [80,87]	70.41 ± 2.38	47.32 ± 17.79	3.74 ± 0.76	12.23 ± 0.65	n.d.a	8.83 ± 4.15	9.97 ± 0.74	4.55 ± 1.05	4.82 ± 1.15	n.d.a	n.d.a	n.d.a	n.d.a
*Ulva intestinalis* [37,78]	31.28 ± 9.77	21.28 ± 2.80	18.45 ± 25.67	10.42 ± 8.73	n.d.a	n.d.a	32.58 ± 9.30	n.d.a	n.d.a	n.d.a	n.d.a	n.d.a	0.87 ± 0.74
*Ulva lactuca* [35,37,79,81,83,88]	54.95 ± 26.77	33.39 ± 12.87	2.21 ± 1.32	15.45 ± 7.71	3.31 ± 3.64	8.88 ± 4.49	27.62 ± 19.64	5.54 ± 3.57	5.64 ± 6.03	2.50 ± 4.03	1.69 ± 1.12	0.65 ± 0.63	0.77 ± 0.48
*Ulva ohnoi* [86]	54.10 ± 0.60	41.30 ± 0.40	1.70 ± 0.17	11.50 ± 0.90	3.90 ± 0.04	0.70 ± 0.10	2.60 ± 0.03	11.60 ± 0.10	0.90 ± 0.06	0.70 ± 0.02	1.50 ± 0.04	2.30 ± 0.10	0.70 ± 0.00
*Ulva reticulata* [78]	43.01 ± 0.46	44.66 ± 0.38	23.96 ± 0.38	6.88 ± 0.42	n.d.a	n.d.a	25.75 ± 0.52	n.d.a	n.d.a	6.01 ± 0.63	n.d.a	n.d.a	0.11 ± 0.02
*Ulva rigida* [85]	24.10 ± 1.40	20.20 ± 0.40	2.90 ± 1.00	13.00 ± 0.30	1.30 ± 0.10	9.50 ± 0.30	62.90 ± 1.10	1.50 ± 0.10	10.90 ± 0.40	1.20 ± 0.10	1.40 ± 0.10	4.10 ± 0.10	0.33
*Ulva tepida* [86]	44.50 ± 0.80	35.70 ± 0.60	1.30 ± 0.13	20.70 ± 0.29	1.40 ± 0.02	15.70 ± 0.10	35.40 ± 0.50	8.60 ± 0.10	13.40 ± 0.20	0.40 ± 0.10	0.80 ± 0.10	1.80 ± 0.10	0.50 ± 0.00
OCHROPHYTA	38.00 ± 13.31	25.77 ± 10.22	8.19 ± 12.98	21.36 ± 7.77	3.89 ± 3.70	15.25 ± 8.64	36.87 ± 15.84	6.21 ± 3.84	4.41 ± 2.70	11.61 ± 6.94	7.80 ± 6.92	2.15 ± 4.18	1.28 ± 0.83
Acinetosporaceae													
*Feldmannia indica* [86]	61.30 ± 0.10	49.00 ± 0.20	1.90 ± 0.03	21.10 ± 0.04	4.40 ± 0.10	16.00 ± 0.04	17.50 ± 0.10	3.70 ± 0.01	5.10 ± 0.02	4.00 ± 0.02	1.50 ± 0.02	0.1 ± 0.00	1.0 ± 0.00
Agaraceae													
*Costaria costata* [78]	37.46 ± 2.02	23.88 ± 1.12	30.84 ± 1.13	15.92 ± 1.46	n.d.a	n.d.a	36.59 ± 2.62	n.d.a	n.d.a	22.00 ± 1.92	n.d.a	n.d.a	1.40 ± 0.18
Alariaceae	28.67 ± 10.17	20.25 ± 7.03	10.49 ± 18.54	15.75 ± 8.92	0.88 ± 0.55	13.65 ± 9.24	54.94 ± 17.93	6.43 ± 2.40	5.17 ± 3.49	10.70 ± 5.30	11.93 ± 4.34	n.d.a	0.677 ± 0.23
*Alaria esculenta* [83]	37.4 ± 0.4	26.9 ± 0.4	1.7 ± 0.0	25.4 ± 0.5	1.5 ± 0.0	23.9 ± 0.5	33.2 ± 0.2	8.2 ± 0.2	5.1 ± 0.0	4.6 ± 0.0	7.1 ± 0.1	n.d.a	0.6 ± 0.0
*Alaria marginata* [81]	n.d.a	14.9	1.1	n.d.a	1.8	11.1	64.7	3.7	9.4	14.9	15.5	n.d.a	n.d.a
*Undaria pinnatifida* [78,89]	24.31 ± 9.63	19.61 ± 8.64	19.58 ± 26.48	10.93 ± 4.43	1.66 ± 0.06	n.d.a	60.94 ± 18.03	n.d.a	n.d.a	n.d.a	n.d.a	8.55 ± 0.37	0.84 ± 0.22
Chordariaceae													
*Myriogloea sciurus* [90] *	65.50	46.30	3.80	26.70	6.50	19.70	7.80	3.70	1.90	1.30	0.90	0.00	1.80
Cladostephaceae													
*Cladostephus spongiosus* [82]	31.74 ± 0.35	21.33 ± 0.35	1.15 ± 0.03	12.15 ± 0.33	5.72 ± 0.28	6.43 ± 0.18	56.11 ± 0.35	23.14 ± 0.26	3.10 ± 0.03	16.43 ± 0.13	11.46 ± 0.10	n.d.a	3.89
Dictyotaceae	38.53 ± 13.50	24.83 ± 9.14	1.87 ± 0.92	21.01 ± 4.75	8.32 ± 6.03	12.21 ± 5.04	24.63 ± 11.11	5.34 ± 3.49	2.27 ± 0.90	9.31 ± 6.57	5.31 ± 4.08	0.42 ± 0.36	1.51 ± 1.07
*Dictyopteris jolyana* [84]	n.d.a	21.05 ± 1.05	0.91 ± 0.05	n.d.a	n.d.a	17.67 ± 0.90	n.d.a	7.89 ± 0.40	2.69 ± 0.13	n.d.a	n.d.a	n.d.a	n.d.a
*Dictyota dichotoma* [36]	25.98 ± 2.52	13.36 ± 1.56	3.52 ± 0.16	14.53 ± 1.21	3.71 ± 0.36	10.82 ± 0.85	18.26 ± 1.36	1.85 ± 0.13	1.68 ± 0.36	7.54 ± 0.60	4.77 ± 0.21	n.d.a	0.70
*Dictyota dichotoma* [90]	45.98 ± 0.47	24.75 ± 0.32	2.85 ± 0.08	24.28 ± 0.13	15.49 ± 0.09	8.49 ± 0.13	29.74 ± 0.67	5.55 ± 0.02	2.63 ± 0.20	11.46 ± 0.59	6.57 ± 0.22	n.d.a	3.52
*Dictyota spiralis* [90]	40.20 ± 0.31	21.69 ± 0.22	2.43 ± 0.04	29.34 ± 0.14	19.58 ± 0.12	9.47 ± 0.10	30.46 ± 0.28	6.05 ± 0.10	3.38 ± 0.05	18.40 ± 0.21	n.d.a	n.d.a	n.d.a
*Padina boergesenii* [86]	68.00 ± 0.40	49.20 ± 0.30	2.30 ± 0.10	20.50 ± 0.80	3.20 ± 0.30	16.80 ± 0.80	11.40 ± 0.50	3.30 ± 0.10	2.20 ± 0.1	2.0 ± 0.1	0.3 ± 0.02	n.d.a	1.4 ± 0.03
*Padina pavonia* [36,91]	31.88 ± 1.17	21.45 ± 4.24	2.10 ± 0.61	21.86 ± 3.74	6.33 ± 1.16	14.60 ± 1.27	21.30 ± 2.66	6.75 ± 7.33	2.04 ± 2.24	7.56 ± 1.53	3.98 ± 0.23	0.28 ± 0.08	1.07 ± 0.51
*Spatoglossum schroederi* [84]	n.d.a	30.44 ± 1.19	1.16 ± 0.25	n.d.a	n.d.a	18.26 ± 0.86	n.d.a	3.93 ± 0.18	2.01 ± 0.06	n.d.a	n.d.a	n.d.a	n.d.a
*Stypopodium schimperi* [91]	28.86 *	21.88 ± 0.05	0.53 ± 0.05	18.36 *	3.82 ± 0.02	13.9 ± 0.19	17.45 *	1.26 ± 0.01	2.40 ± 0.02	1.33 ± 0.03	4.07 ± 0.1	0.15 ± 0.0	0.53
*Taonia atomaria* [82]	35.47 ± 1.02	25.41 ± 0.97	1.04 ± 0.21	17.34 ± 0.61	8.09 ± 0.10	8.09 ± 0.71	47.19 ± 0.65	10.08 ± 0.32	1.71 ± 0.08	18.64 ± 0.11	13.55 ± 0.55	0.84 ± 0.03	2.28
*Zonaria tournefortii* [84]	n.d.a	22.49 ± 1.17	1.69 ± 0.09	n.d.a	n.d.a	1.69 ± 0.09	n.d.a	5.40 ± 0.39	n.d.a	n.d.a	n.d.a	n.d.a	n.d.a
Fucaceae	25.14 ± 2.87	13.46 ± 3.44	1.45 ± 1.14	33.21 ± 11.11	1.45 ± 0.47	28.91 ± 11.90	42.02 ± 10.09	8.58 ± 2.45	4.40 ± 2.70	12.56 ± 4.49	7.68 ± 3.22	n.d.a	1.84 ± 0.70
*Ascophyllum nodosum* [53]	25.14 ± 0.49	13.42 ± 0.46	0.76 ± 0.01	31.15 ± 0.23	2.24 ± 0.01	27.83 ± 0.26	43.47 ± 0.54	7.47 ± 0.12	4.45 ± 0.03	17.25 ± 0.26	7.24 ± 0.08	0.00 ± 0.00	2.62 ± 0.01
*Fucus distichus* [81] *	n.d.a	19.60	0.80	n.d.a	2.20	16.70	49.10	7.70	7.90	14.70	10.90	n.d.a	n.d.a
*Fucus vesiculosus* [53,83,84,85]	26.25 ± 2.64	11.65 ± 0.35	1.75 ± 1.61	30.91 ± 14.02	1.09 ± 0.09	29.38 ± 14.43	41.86 ± 14.01	7.64 ± 1.08	3.69 ± 3.50	11.63 ± 5.98	7.98 ± 3.71	n.d.a	1.64 ± 0.39
Himanthaliaceae													
*Himanthalia elongata* [92]	39.06 ± 2.11	32.53 ± 1.61	0.68 ± 0.15	22.75 ± 2.26	2.79 ± 0.25	19.69 ± 2.01	38.16 ± 7.84	4.39 ± 0.34	8.79 ± 0.71	10.69 ± 1.30	5.50 ± 1.78	n.d.a	0.81
Hormosiraceae													
*Hormosira banksii* [90] *	40.60	27.50	1.50	24.60	3.70	18.70	34.80	5.50	7.40	13.20	5.80	0.00	1.50
Laminariaceae	36.16 ± 9.97	23.53 ± 5.17	5.93 ± 12.35	21.65 ± 7.89	3.26 ± 3.92	21.24 ± 9.70	38.91 ± 11.58	6.03 ± 2.46	4.72 ± 2.09	11.07 ± 6.32	8.04 ± 3.60	n.d.a	1.06 ± 0.96
*Ecklonia radiate* [90] *	50.70	27.90	1.80	33.00	11.40	20.20	16.30	3.00	1.80	8.70	2.30	0.00	3.00
*Hedophyllum sessile* [81] *	n.d.a	20.10	2.50	n.d.a	1.20	40.70	27.10	6.50	3.30	9.70	3.10	n.d.a	n.d.a
*Laminaria dentigera* [81] *	n.d.a	29.60	2.40	n.d.a	2.30	20.00	40.20	5.10	4.70	9.80	10.40	n.d.a	n.d.a
*Laminaria digitata* [83,93]	36.03 ± 6.41	25.79 ± 5.78	1.82 ± 1.44	16.50 ± 3.10	3.12 ± 3.13	13.38 ± 6.24	44.15 ± 2.04	7.24 ± 3.19	6.79 ± 1.00	7.55 ± 0.78	11.56 ± 0.22	n.d.a	n.d.a
*Laminaria hyperborea* [83]	33.70 ± 0.70	23.30 ± 0.40	1.30 ± 0.50	26.50 ± 4.20	n.d.a	26.50 ± 4.20	34.20 ± 3.20	5.00 ± 0.20	3.80 ± 0.40	7.60 ± 0.80	9.50 ± 1.00	n.d.a	0.60 ± 0.00
*Macrocystis integrifolia* [81] *	n.d.a	16.40	1.10	n.d.a	2.60	12.20	52.50	4.30	7.50	14.70	8.70	n.d.a	n.d.a
*Postelsia palmaeformis* [81] *	n.d.a	26.10	2.20	n.d.a	1.20	23.60	40.30	9.90	6.50	8.20	7.20	n.d.a	n.d.a
*Saccharina japonica* [78]	24.35 ± 1.18	16.79 ± 0.75	38.83 ± 0.79	15.76 ± 0.64	n.d.a	n.d.a	51.28 ± 0.61	n.d.a	n.d.a	26.93 ± 0.18	n.d.a	n.d.a	0.65 ± 0.09
Lessoniaceae	n.d.a	24.37 ± 3.55	n.d.a	n.d.a	n.d.a	16.62 ± 3.33	n.d.a	4.58 ± 1.50	n.d.a	8.41 ± 7.23	6.20 ± 3.80	n.d.a	0.52 ± 0.0
*Ecklonia kurome* [94]	n.d.a	24.43 ± 0.64	n.d.a	n.d.a	6.93 ± 0.17	16.07 ± 0.14	n.d.a	4.74 ± 0.19	4.62 ± 0.38	1.03 ± 0.18	6.40 ± 0.26	1.03 ± 0.18	0.52
*Egregia menziesii* [81] *	n.d.a	20.80	1.60	n.d.a	1.50	13.60	54.90	6.00	9.30	15.50	9.90	n.d.a	n.d.a
Monodopsidaceae													
*Nannochloropsis oceanica* [95]	33.00 ± 5.10	22.60 ± 2.00	6.30 ± 3.20	27.40 ± 0.80	0.10 ± 0.00	0.50 ± 0.10	39.70 ± 3.40	4.10 ± 0.30	3.20 ± 0.30	5.30 ± 0.60	30.80 ± 2.40	n.d.a	0.3 ± 0.0
Phyllariaceae													
*Saccorhiza polyschides* [92]	48.54 ± 0.85	42.14 ± 0.58	0.65 ± 0.02	29.74 ± 0.92	9.06 ± 0.40	15.01 ± 0.27	21.70 ± 0.80	2.84 ± 0.08	3.36 ± 0.09	6.11 ± 0.33	3.01 ± 0.11	n.d.a_	0.71
Ralfsiaceae													
*Analipus japonicus* [81] *	n.d.a	19.90	0.70	n.d.a	0.70	10.90	59.10	8.40	8.10	14.90	13.20	n.d.a	n.d.a
Sargassaceae	39.30 ± 13.69	28.11 ± 10.45	16.24 ± 15.37	18.40 ± 5.00	3.46 ± 2.42	10.98 ± 3.99	39.18 ± 16.51	5.74 ± 2.05	5.48 ± 3.14	14.78 ± 7.50	8.19 ± 9.60	3.22 ± 5.28	1.33 ± 0.69
*Bifurcaria bifurcata* [53,68]	38.85 ± 13.06	23.71 ± 8.99	6.08 ± 6.13	21.35 ± 5.88	2.67 ± 0.23	12.36 ± 0.35	41.81 ± 7.20	2.57 ± 0.92	2.13 ± 2.41	14.76 ± 0.68	4.40 ± 0.44	11.10 ± 1.13	1.31 ± 0.14
*Cystoseira Hakodatensi* [94]	n.d.a	18.49 ± 0.30	n.d.a	n.d.a	0.63 ± 0.08	11.08 ± 0.24	n.d.a	6.95 ± 0.15	6.87 ± 0.18	16.59 ± 0.11	12.96 ± 0.18	n.d.a	1.32
*Cystoseria osmundacea* [81] *	n.d.a	22.5	0.8	n.d.a	2.6	11.8	54.1	5.8	10.6	19.1	5.5	n.d.a	n.d.a
*Hizikia fusiforme* [89]	28.10 ± 4.30	26.80 ± 3.84	0.76 ± 0.31	13.40 ± 6.40	0.15 ± 0.07	7.68 ± 4.22	57.0 ± 11.6	3.56 ± 1.45	0.56 ± 0.21	5.30 ± 1.98	42.4 ± 11.88	n.d.a	0.31 ± 0.10
*Sargassum aquifolium* [79,86]	61.21 ± 0.52	49.12 ± 0.51	3.23 ± 0.01	26.01 ± 0.30	4.63 ± 0.11	19.80 ± 0.21	12.91 ± 0.33	3.30 ± 0.11	1.72 ± 0.04	4.31 ± 0.10	0.90 ± 0.12	n.d.a	2.9 ± 0.03
*Sargassum crassifolium* [94]	n.d.a	25.14 ± 2.90	n.d.a	n.d.a	2.64 ± 0.38	11.17 ± 0.41	n.d.a	6.71 ± 0.36	5.36 ± 0.33	8.46 ± 0.19	1.79 ± 0.57	n.d.a	1005
*Sargassum fusiforme* [78]	27.62 ± 0.58	24.80 ± 0.52	27.01 ± 0.43	11.95 ± 0.22	n.d.a	n.d.a	47.32 ± 1.08	n.d.a	n.d.a	30.38 ± 0.45	n.d.a	n.d.a	0.57 ± 0.01
*Sargassum horneri* [78,94]	26.98 ± 0.00	25.24 ± 1.91	28.90 ± 2.33	14.24 ± 0.00	n.d.a	n.d.a	49.00 ± 0.00	n.d.a	n.d.a	23.04 ± 0.52	n.d.a	n.d.a	0.99 ± 0.30
*Sargassum Ilicifolium* [85]	55.20 ± 3.33	46.10 ± 2.80	1.90 ± 0.46	27.50 ± 2.58	8.20 ± 0.64	18.80 ± 2.16	17.40 ± 0.68	7.90 ± 0.42	5.90 ± 0.72	1.00 ± 0.12	1.90 ± 0.22	0.2 ± 0.11	1.1
*Sargassum muticum* [68]	32.62 ± 0.92	24.18 ± 0.48	3.29 ± 0.33	16.36 ± 0.49	2.34 ± 0.43	7.91 ± 0.14	51.02 ± 4.79	5.61 ± 0.26	7.07 ± 0.07	12.53 ± 0.72	9.71 ± 0.34	n.d.a	0.74 ± 0.03
*Sargassum oligocystum* [85]	61.63 ± 6.17	41.56 ± 3.27	2.86 ± 0.32	17.71 ± 1.12	n.d.a	17.71 ± 1.12	10.13 ± 0.79	6.33 ± 0.23	3.80 ± 0.56	n.d.a	n.d.a	n.d.a	n.d.a
*Sargassum pallidum* [96]	27.30 ± 1.00	21.80 ± 0.90	0.30 ± 0.00	17.20 ± 0.70	7.00 ± 0.30	7.80 ± 0.40	55.50 ± 2.90	10.90 ± 0.70	1.20 ± 0.0	17.10 ± 0.80	5.40 ± 0.20	0.1 ± 0.0	1.94
*Sargassum siliquastrum* [94]	n.d.a	4.78 ± 0.90	n.d.a	n.d.a	1.68 ± 0.19	8.62 ± 0.18	n.d.a	5.51 ± 0.14	9.48 ± 0.23	16.95 ± 0.07	8.82 ± 0.30	n.d.a	1.05
*Sargassum vulgare* [82]	42.34 ± 0.28	31.23 ± 0.24	1.62 ± 0.11	19.03 ± 0.14	8.61 ± 0.11	7.40 ± 0.06	38.63 ± 0.32	7.59 ± 0.02	n.d.a	18.64 ± 0.04	8.60 ± 0.12	1.50 ± 0.07	2.82
*Turbinaria ornate* [94]	n.d.a	30.24 ± 0.79	n.d.a	n.d.a	3.54 ± 0.13	9.53 ± 0.19	n.d.a	5.66 ± 0.20	8.27 ± 0.50	15.06 ± 0.43	4.21 ± 0.20	n.d.a	0.95
Scytosiphonaceae	44.74 ± 21.01	33.26 ± 15.79	2.60 ± 1.62	20.34 ± 9.09	2.53 ± 0.90	17.59 ± 8.30	19.95 ± 10.44	2.62 ± 0.45	1.68 ± 0.47	3.66 ± 3.37	3.90 ± 2.41	6.27 ± 7.01	0.89 ± 0.53
*Colpomenia sinuosa* [36,78]	39.89 ± 16.98	29.37 ± 12.20	1.90 ± 2.41	23.27 ± 13.61	3.08 ± 0.87	19.89 ± 12.31	21.91 ± 9.36	2.89 ± 0.28	1.55 ± 0.34	3.48 ± 4.36	5.18 ± 0.31	6.27 ± 8.59	0.74 ± 0.36
Seirococcaceae													
*Phyllospora comosa* [90] *	42.00	25.40	1.70	20.80	6.10	13.70	37.20	7.00	3.90	21.10	3.90	0.00	3.80
Stypocaulaceae													
*Halopteris scoparia* [82]	34.89 ± 0.51	24.36 ± 0.45	1.92 ± 0.10	14.09 ± 0.28	5.47 ± 0.09	8.23 ± 0.34	51.01 ± 0.98	20.35 ± 0.14	n.d.a	13.96 ± 0.36	14.39 ± 0.25	0.99 ± 0.86	2.32
RHODOPHYTA	45.29 ± 15.54	35.23 ± 14.64	4.36 ± 4.94	16.86 ± 8.51	4.56 ± 4.87	7.50 ± 4.81	31.29 ± 17.70	1.90 ± 1.27	1.98 ± 3.11	10.88 ± 10.98	23.33 ± 16.63	6.91 ± 16.76	1.68 ± 3.14
Alsidieae													
*Alsidium seaforthii* [84]	n.d.a	21.65 ± 0.47	2.63 ± 0.20	n.d.a	n.d.a	10.40 ± 0.87	n.d.a	4.68 ± 1.05	n.d.a	n.d.a	n.d.a	n.d.a	n.d.a
Bangiaceae	n.d.a	n.d.a	3.87 ± 1.30	n.d.a	10.75 ± 9.25	4.37 ± 2.78	n.d.a	n.d.a	n.d.a	6.13 ± 6.25	28.92 ± 12.19	n.d.a	n.d.a
*Porphyra columbina* [97]	n.d.a	21.55 ± 0.70	3.55 ± 0.15	n.d.a	3.13 ± 0.27	8.50 ± 0.54	n.d.a	3.41 ± 0.09	11.40 ± 1.15	15.52 + 0.08	28.36 + 0.33	n.d.a	n.d.a
*Porphyra dioica* [85,98]	34.32 ± 4.49	25.71 ± 3.40	3.53 ± 1.93	15.47 ± 9.94	10.78 ± 11.90	2.85 ± 0.63	48.78 ± 12.42	1.67 ± 0.04	1.95 ± 0.07	3.16 ± 0.19	33.42 ± 18.27	n.d.a	1.22 ± 1.49
Bonnemaisoniaceae													
*Asparagopsis armata* [82]	81.3 ± 0.56	53.21 ± 0.52	2.81 ± 0.16	13.99 ± 0.94	4.87 ± 0.92	9.12 ± 0.31	4.70 ± 0.38	n.d.a	n.d.a	1.79 ± 0.34	2.90 ± 0.15	n.d.a	0.62
Callithamniaceae													
*Callitkamnion corymbosum* [99]	34.50 *	25.90 ± 0.20	2.20 ± 0.10	35.80 *	3.40 ± 0.10	6.00 ± 0.20	29.10 *	2.60 ± 0.10	3.50 ± 0.20	12.30 ± 0.30	43.40 ± 0.80	n.d.a	n.d.a
Ceramiaceae	49.16 ± 4.27	36.29 ± 4.75	1.37 ± 0.06	19.09 ± 0.46	12.42 ± 0.61	5.48 ± 0.61	21.95 ± 18.73	1.41 ± 0.32	1.52 ± 1.41	4.43 ± 0.93	14.12 ± 18.6	0.69 ± 0.15	1.57 ± 1.81
*Spyridia filamentosa* [91]	52.18 *	39.65 ± 0.83	1.41 ± 0.06	19.53 *	12.09 ± 0.3	5.04 ± 0.03	8.70 *	1.18 ± 0.07	0.52 ± 0.06	5.09 ± 0.82	0.99 ± 0.14	0.69 ± 0.15	2.85
*Bornetia secundiflora* [82]	46.14 ± 0.82	32.93 ± 0.75	1.33 ± 0.22	18.66 ± 0.46	12.75 ± 0.26	5.91 ± 0.45	35.20 ± 0.66	1.64 ± 0.10	2.53 ± 0.02	3.78 ± 0.10	27.26 ± 0.64	n.d.a	0.29
Corallinaceae													
*Jania rubens* [91] *	40.08 *	29.85 ± 3.15	0.75 ± 0.08	12.52 *	5.15 ± 0.34	4.36 ± 0.18	34.14 *	1.05 ± 0.03	0.10 ± 0.04	4.43 ± 0.07	24.47 ± 0.94	0.53 ± 0.05	0.24
Dasyaceae													
*Dasya rigidula* [91]	60.43 *	45.15 ± 0.22	3.20 ± 0.36	23.43 *	10.59 ± 0.13	11.93 ± 0.84	8.57 *	2.02 ± 0.15	0.34 ± 0.03	1.85 ± 0.17	4.36 ± 0.35	n.d.a	0.82
Delesseriaceae	n.d.a	34.40 ± 7.07	n.d.a	n.d.a	4.10 ± 3.67	12.75 ± 10.11	n.d.a	1.60 ± 1.41	1.15 ± 1.34	14.85 ± 6.43	19.15 ± 18.87	n.d.a	n.d.a
*Apoglossum ruscifofium* [99]	21.1 *	39.40 ± 0.40	n.d.a	29.9 *	6.70 ± 0.30	19.90 ± 0.40	42.4 *	2.60 ± 0.l0	2.10 ± 0.30	10.30 ± 0.50	5.80 ± 0.30	n.d.a	n.d.a
*Cryptopleura violaceae* [81] *	n.d.a	29.40	0.90	n.d.a	1.50	4.40	n.d.a	0.60	0.50	19.40	32.50	n.d.a	n.d.a
Endocladiaceae													
*Gloiopeltis furcata* [78]	26.97 ± 1.45	23.34 ± 1.31	19.36 ± 0.11	18.05 ± 0.23	n.d.a	n.d.a	45.33 ± 2.36	n.d.a	n.d.a	44.89 ± 2.24	n.d.a	n.d.a	0.19 ± 0.00
Florideophyceae													
*Ceramium strictum* [99]	34.80 *	24.00 ± 0.60	3.30 ± 0.20	30.30 *	7.30 ± 0.30	18.30 ± 0.20	34.90 *	2.00 ± 0.10	15.10 ± 0.30	3.90 ± 0.10	n.d.a	n.d.a	n.d.a
Halymeniaceae	n.d.a	27.93 ± 5.50	2.44 ± 2.59	n.d.a	0.92 ± 0.56	8.04 ± 1.21	n.d.a	1.89 ± 1.81	1.13 ± 1.08	18.70 ± 5.33	26.95 ± 5.71	n.d.a	n.d.a
*Grateloupia turuturu* [100]	37.49 ± 5.91	22.03 ± 0.98	5.44 ± 0.66	16.66 ± 1.29	1.58 ± 0.14	7.44 ± 0.24	49.51 ± 6.18	3.99 ± 0.28	2.13 ± 0.18	12.91 ± 0.56	20.86 ± 0.56	n.d.a	n.d.a
*Halymenia brasiliana* [84]	n.d.a	35.32 ± 1.08	n.d.a	n.d.a	n.d.a	6.62 ± 0.34	n.d.a	n.d.a	2.01 ± 1.74	n.d.a	n.d.a	n.d.a	n.d.a
*Prionitis lanceolata* [81] *	n.d.a	27.70	1.00	n.d.a	0.60	6.30	n.d.a	1.00	1.10	19.80	32.20	n.d.a	n.d.a
*Prionitis linearis* [81] *	n.d.a	26.70	0.90	n.d.a	0.60	6.20	n.d.a	0.70	0.60	23.40	27.80	n.d.a	n.d.a
Hypneaceae	65.76 ± 2.32	40.43 ± 27.39	3.35 ± 1.77	16.00 ± 10.74	8.50 ± 0.14	5.95 ± 3.47	6.97 ± 3.47	3.27 ± 3.15	0.89 ± 0.83	5.4 ± 0.42	1.00 ± 0.04	0.70 ± 0.13	2.70
*Hypnea musciformis* [35]	64.12 ± 20.82	21.06 ± 3.88	4.60 ± 0.58	8.41 ± 1.64	n.d.a	8.41 ± 1.64	4.75 ± 3.84	3.27 ± 2.43	1.48 ± 1.41	n.d.a	n.d.a	n.d.a	n.d.a
*Hypnea valentiae* [35]	67.40 ± 0.31	59.80 ± 1.09	2.10 ± 0.37	23.60 ± 0.68	8.50 ± 0.14	3.50 ± 0.28	9.20 ± 0.80	n.d.a	0.30 ± 0.12	5.4 ± 0.42	1.00 ± 0.04	0.70 ± 0.13	2.70
Gigartinaceae	38.33 ± 6.48	31.89 ± 5.27	7.04 ± 6.95	11.06 ± 3.01	0.56 ± 0.25	9.55 ± 3.62	43.84 ± 12.60	1.32 ± 0.45	0.37 ± 0.30	21.60 ± 14.97	38.69 ± 6.10	n.d.a	0.67 ± 0.12
*Chondrus crispus* [98]	32.44 ± 0.90	27.33 ± 0.50	2.71 ± 0.05	8.82 ± 0.24	0.58 ± 0.08	6.06 ± 0.03	56.42 ± 1.29	1.58 ± 0.05	0.72 ± 0.01	19.85 ± 0.82	33.47 ± 0.21	n.d.a	0.66 ± 0.03
*Chondrus yendoi* [78]	37.28 ± 0.62	33.12 ± 0.48	12.60 ± 0.23	9.89 ± 0.19	n.d.a	n.d.a	43.91 ± 0.85	n.d.a	n.d.a	42.35 ± 0.89	n.d.a	n.d.a	0.81 ± 0.03
*Gigartina harveyana* [81] *	n.d.a	28.60	1.70	n.d.a	0.80	12.50	n.d.a	1.60	0.60	10.40	37.20	n.d.a	n.d.a
*Iridaea cordata* [81] *	n.d.a	29.90	1.80	n.d.a	0.30	8.50	n.d.a	0.80	0.50	5.30	45.40	n.d.a	n.d.a
*Mazzaella japonica* [78]	45.29 ± 0.51	40.50 ± 0.38	16.42 ± 0.05	14.49 ± 0.61	n.d.a	n.d.a	31.21 ± 0.72	n.d.a	n.d.a	30.12 ± 0.29	n.d.a	n.d.a	0.56 ± 0.01
Gracilariaceae	44.18 ± 21.02	47.83 ± 26.16	3.72 ± 2.97	19.41 ± 9.98	2.31 ± 2.34	5.79 ± 5.67	28.44 ± 18.25	1.00 ± 0.78	2.29 ± 2.82	8.45 ± 12.01	3.29 ± 2.02	n.d.a	2.38 ± 2.71
*Gracilaria changii* [101]	7.53 ± 1.72	4.28 ± 0.58	1.82 ± 0.27	38.30 ± 7.20	0.28 ± 0.04	0.15 ± 0.07	51.20 ± 6.78	0.59 ± 0.08	0.17 ± 0.02	0.26 ± 0.02	n.d.a	48.36 ± 6.76	0.02 ± 0.00
*Gracilaria corticata* [35]	58.7 ± 3.75	43.6 ± 3.14	2.2 ± 0.72	22.0 ± 3.65	6.9 ± 0.40	13.8 ± 3.30	19.1 ± 2.86	n.d.a	7.7 ± 1.88	5.2 ± 0.88	n.d.a	n.d.a	1.4
*Gracilaria domingensis* [84]	n.d.a	68.16 ± 1.15	n.d.a	n.d.a	n.d.a	8.38 ± 0.10	n.d.a	n.d.a	n.d.a	n.d.a	n.d.a	n.d.a	n.d.a
*Gracilaria edulis* [102]	n.d.a	84.60	1.24	n.d.a	0.38	0.71	n.d.a	0.16	n.d.a	0.67	n.d.a	n.d.a	n.d.a
*Gracilaria folifera* [102]	n.d.a	81.28	1.51	n.d.a	0.47	1.00	n.d.a	0.21	0.10	0.57	n.d.a	n.d.a	n.d.a
*Gracilaria gracilis* [85]	34.9 ± 0.9	27.1 ± 1.2	4.6 ± 0.8	12.5 ± 0.7	2.8 ± 0.8	9.7 ± 0.4	52.6 ± 1.4	2 ± 0.4	2.7 ± 0.2	35.4 ± 1.5	5.5 ± 0.2	n.d.a	2.47
*Gracilaria salicornia* [88]	48.92 ± 6.83	33.39 ± 8.86	3.04 ± 0.66	16.36 ± 1.54	2.46 ± 0.12	11.72 ± 2.01	17.30 ± 1.18	1.45 ± 0.38	1.65 ± 0.04	8.05 ± 1.98	1.53 ± 0.27	n.d.a	1.2
*Gracilariopsis longissima* [78]	47.78 ± 2.36	46.44 ± 0.91	10.16 ± 0.50	11.18 ± 1.73	n.d.a	n.d.a	13.90 ± 0.64	n.d.a	n.d.a	15.21 ± 0.69	n.d.a	n.d.a	7.69 ± 1.58
*Gracilariopsis longissima* [103]	67.30 ± 3.10	41.67 ± 1.81	5.21 ± 0.31	16.16 ± 1.15	2.91 ± 0.10	0.93 ± 0.01	16.54 ± 1.24	1.60 ± 0.20	1.46 ± 0.00	2.26 ± 0.11	2.84 ± 0.33	1.85 ± 0.21	1.50
Palmariaceae													
*Palmaria palmata* [83,85,99,104]	44.12 ± 2.06	30.25 ± 5.22	5.37 ± 6.17	6.32 ± 2.06	1.68 ± 0.30	3.87 ± 1.15	44.86 ± 8.79	1.09 ± 0.70	n.d.a	0.92 ± 0.23	41.17 ± 9.10	n.d.a	0.045 ± 0.06
Plocamiaceae	74.10 ± 4.10	17.20 ± 17.68	0.65 ± 0.35	4.30 ± 0.10	1.25 ± 1.63	1.05 ± 1.06	20.40 ± 4.40	0.35 ± 0.35	0.50	4.40 ± 5.37	19.00 ± 25.74	n.d.a	0.70 ± 0.10
*Plocamium brasiliense* [105]	74.10 ± 4.10	4.70 ± 0.10	0.40 ± 0.00	4.30 ± 0.10	0.10 ± 0.00	0.30 ± 0.00	20.40 ± 4.40	0.10 ± 0.00	n.d.a	0.60 ± 0.30	0.80 ± 0.10	n.d.a	0.70 ± 0.10
*Plocamium violaceum* [81] *	n.d.a	29.70	0.90	n.d.a	2.40	1.80	n.d.a	0.60	0.50	8.20	37.20	n.d.a	n.d.a
Pterocladiaceae													
*Pterocladiella capillacea* [82]	60.62 ± 0.65	47.94 ± 0.64	2.21 ± 0.04	8.45 ± 0.10	3.15 ± 0.09	5.30 ± 0.01	30.94 ± 0.40	2.27 ± 0.05	1.94 ± 0.10	10.33 ± 0.09	15.26 ± 0.13	n.d.a	0.60
Rhizophyllidaceae													
*Ochtodes secundiramea* [105]	66.10 ± 1.30	6.10 ± 0.10	0.40 ± 0.10	21.50 ± 0.10	0.10 ± 0.00	2.50 ± 0.10	8.30 ± 0.10	0.20 ± 0.00	n.d.a	n.d.a	0.80 ± 0.00	n.d.a	4.00 ± 0.0
Rhodomelaceae	45.66 ± 18.37	32.95 ± 8.64	4.65 ± 6.88	17.81 ± 4.97	4.94 ± 3.91	9.54 ± 2.77	27.64 ± 17.97	3.15 ± 1.41	1.02 ± 1.83	11.45 ± 5.18	22.07 ± 12.34	2.44 ± 0.23	0.52 ± 0.19
*Acanthophora spicifera* [102]	n.d.a	40.27	1.43	n.d.a	1.43	10.21	n.d.a	1.37	0.79	10.19	6.18	n.d.a	n.d.a
*Alsidium triquetrum* [84]	n.d.a	27.36 ± 0.33	n.d.a	n.d.a	n.d.a	7.50 ± 0.31	n.d.a	3.24 ± 0.10		n.d.a	n.d.a	n.d.a	n.d.a
*Chondria crassicaulis* [78]	36.88 ± 1.23	38.41 ± 1.54	19.93 ± 1.20	20.49 ± 0.88	n.d.a	n.d.a	28.15 ± 1.37	n.d.a	n.d.a	18.50 ± 0.69	n.d.a	n.d.a	0.42 ± 0.03
*Laurencia intermedia* [88]	71.04 ± 0.49	46.88 ± 0.37	2.90 ± 0.07	11.22 ± 0.13	n.d.a	11.22 ± 0.13	3.07 ± 0.06	2.70 ± 0.19	0.37 ± 0.15	n.d.a	n.d.a	n.d.a	n.d.a
*Laurencia papillosa* [91]	46.14	35.27 ± 0.79	1.96 ± 0.07	16.93	8.64 ± 0.11	8.29 ± 0.22	33.57	5.04 ± 0.15	0.31 ± 0.01	8.17 ± 0.1	18.42 ± 0.83	2.44 ± 0.23	0.75
*Odonthalia floccose* [81] *	n.d.a	28.60	0.80	n.d.a	1.70	5.30	n.d.a	1.40	0.40	14.80	31.60	n.d.a	n.d.a
*Osmundaria obtusiloba* [84]	n.d.a	21.27 ± 2.50	4.67 ± 1.05	n.d.a	n.d.a	9.82 ± 1.69	n.d.a	4.21 ± 1.41	5.86 ± 0.48	n.d.a	n.d.a	n.d.a	n.d.a
*Vertebrata lanosa* [83]	28.60 ± 0.20	25.60 ± 0.10	0.90 ± 0.10	22.60 ± 0.80	8.00 ± 0.00	5.60 ± 1.00	45.80 ± 0.90	4.10 ± 0.10	0.80 ± 0.10	5.60 ± 0.20	32.10 ± 1.00	n.d.a	0.40 ± 0.00
Rhodymeniaceae													
*Botryocladia occidentalis* [84]	n.d.a	53.58 ± 0.78	1.11 ± 1.93	n.d.a	n.d.a	20.09 ± 0.63	n.d.a	n.d.a	n.d.a	n.d.a	n.d.a	n.d.a	n.d.a
Solieriaceae	57.80 ± 3.66	38.20 ± 10.26	9.63 ± 7.20	16.11 ± 10.77	5.05 ± 6.85	3.44 ± 1.73	13.18 ± 9.72	1.23 ± 1.46	0.29 ± 0.27	15.20 ± 7.63	2.95 ± 2.89	n.d.a	8.80 ± 9.75
*Eucheuma denticulatum* [88]	53.72 ± 1.55	43.22 ± 17.76	2.30 ± 1.06	4.83 ± 3.43	n.d.a	4.83 ± 3.43	2.75 ± 2.10	2.27 ± 1.50	0.49 ± 0.18	n.d.a	n.d.a	n.d.a	n.d.a
*Solieria chordalis* [75]	58.90 ± 0.10	45.00 ± 0.20	9.90 ± 0.10	26.30 ± 0.10	0.20 ± 0.10	4.00 ± 0.10	14.80 ± 0.10	n.d.a	n.d.a	9.80 ± 0.10	5.00 ± 0.10	n.d.a	1.90 ± 0.10
*Solieria robusta* [91] *	60.80	26.40	16.70	17.20	9.90	1.50	22.00	0.20	0.10	20.60	0.90	0.00	15.70
Spyridiaceae													
*Spyridia clavata* [84]	n.d.a	35.50 ± 3.81	2.45 ± 2.27	n.d.a	n.d.a	13.99 ± 1.45	n.d.a	n.d.a	n.d.a	n.d.a	n.d.a	n.d.a	n.d.a

n.d.a—no data available; *—only mean value available; all data reported as mean ± SD (% of total fatty acids) and if more than one entry available mean and SD was calculated.

## 6. The Anti-Inflammatory Activity of Seaweed Lipids

The anti-inflammatory activity of seaweed derived lipids has been studied using crude lipid extracts, lipid fractions and isolated complex lipids from several seaweeds belonging to the Ochrophyta and Rhodophyta phyla [85,90,98,100,106,107,108,109,110,111,112,113], with only three *Ulva rigida*, *Ulva* sp. and *Codium tomentosum* within the Chlorophyta phylum [85,90] (Table 2, Figure 1). Lipids were generally extracted using a methanol:chloroform ratio of 2:1 [85,98,100,106,107,108,109,111], with only a few studies using different organic solvents [90,109,112,113,114]. The anti-inflammatory properties of seaweeds were assessed by the evaluation of the production of nitric oxide (NO) [85,90,106,108,109,110,111,112] and pro-inflammatory cytokines (IL-6, Il-8, TNF-α, MCP-1) [98,107], expression of several genes involved in inflammatory signalling [98], the activation of NF-κB pathway [107], and the activity of cyclooxygenase-2 (COX-2) [77,80] and inducible NO synthase (iNOS) [108]. The majority of the studies were performed *in chemico* [85,100] and in vitro (murine macrophage RAW 264.7 [85,90,106,108,109,110,111,112] and human THP-1 monocytic cell line [100,109]) with only two in vivo studies using BALB/c mice model [113,115]. There were no studies involving human participants (Table 2).

COX-2 activity and NO levels were the most commonly assessed parameters in the studies evaluating anti-inflammatory properties of seaweed derived lipids. COX-2 is one of the key mediators of inflammatory responses that converts arachidonic acid to prostaglandins and is activated by extracellular and intracellular stimuli, including LPS, cytokines, growth factors, and other pro-inflammatory molecules [115]. COX-2 upregulation and overexpression is associated with inflammation and generated prostaglandins have been reported to act as immunosuppressors [116]. The ability of seaweed lipids to inhibit COX-2 and the same to reduce the synthesis of prostaglandins has been shown [85,100]. Lopes et al. [85] and Da Costa et al. [100] reported the COX-2 inhibition by crude lipid extracts of several seaweed species within the Ochrophyta, Chlorophyta and Rhodophyta phyla, indicating their anti-inflammatory potential. The highest inhibition of COX-2 activity was observed for the lipid extracts (concentration of 500 µg/mL) of *P. palmata* (89.5 ± 0.9%) and *U. rigida* (87.9 ± 0.1%), followed by *P. dioica* (83.6 ± 8.1%) and *C. tomentosum* (82.3 ± 2.2%), Da Costa et al. [100] reported that lipid extracts of *G. turturu* had a slightly lower potential to inhibit COX-2 activity reaching 50% inhibitory effect at a concentration of 33 µg/mL. A significantly lower inhibitory effect was observed for lipid extracts of *F. vesiculosus* with 34.6 ± 7.1% of COX-2 inhibition, and *G. gracilis* showed no inhibition potential of COX-2 activity. It should be noted that the lipid extracts of *G. gracilis* and *F. vesiculosus* were characterised by a higher content of AA and n-6, and lower levels of n-3 fatty acids than other studied seaweeds, which may explain their lower anti-inflammatory potential [85]. Previous studies suggested that n-3 LC-PUFA may reduce the production of cyclo-oxygenase pathway mediators from arachidonic acid. In addition, decreasing arachidonic acid may directly reduce NFκB expression and down-regulate proinflammatory genes, including COX [117].

Several in vitro studies assessed NO production in LPS stimulated murine macrophage RAW 264.7. NO is a signaling molecule that plays a crucial role in inflammation and host defense mechanisms. It may act as anti-inflammatory agent under normal physiological conditions, but NO is a potent proinflammatory mediator in chronic inflammation [118,119,120]. During inflammatory response, pro-inflammatory cytokines stimulate the expression of iNOS in monocyte/macrophages, neutrophil granulocytes, and many other cells and in consequence, large amounts of NO are secreted [119]. Overall, NO plays a crucial role in inflammation and therefore, is a potential target in the treatment of inflammatory diseases. Crude lipid extracts of *Sargassum ilicifolium* obtained from Ujung Genteng Beach–Sukabumi (UGB) and Awur Bay–Jepara (AB) regions and *Gracilaria* sp. were shown to significantly suppress NO production of LPS induced RAW 246.7 cells [106,111]. Similarly, the lipid fractions of *Lobophora* sp. displayed a strong NO inhibitory effect, with the non-polar lipid fraction showing the strongest activity (IC_50_ of 52.10  ±  4.43), followed by total lipid and polar lipid with IC_50_ of 61.09  ±  6.06 and 66.21  ±  6.24 µg/mL, respectively [109]. Moreover, several studies evaluated the anti-inflammatory role of isolated seaweed complex lipids. Banskota et al. [108,110] reported that polar lipids, including sulfoquinovosyl diacylglycerols, phosphatidylglycerols, phospholipid, and galactolipids isolated from *Palmaria palmata* and EPA, AA and galactolipids from *Chondrus crispus* possessed strong and dose-dependent NO inhibitory activity through down-regulation of inducible nitric oxide synthase (iNOS)**.** However, it was observed that galactolipids have stronger NO inhibitory activity as compared to free polyunsaturated fatty acids, including EPA and AA, suggesting that the entire polar lipid structure may be essential for potent anti-inflammatory action [108]. On the other hand, it was suggested that the PUFA side chains of galactolipids are essential for NO inhibition [121]. Similar findings were reported by Lopes et al. [112] who observed that galactolipids isolated from *Fucus spiralis* inhibit NO release by LPS stimulated RAW 264.7 macrophages. However, the monoacylglycerol, composed of a glycerol moiety linked to oleic acid, demonstrated slightly lower capacity to inhibit NO production by macrophages than the 1:1 mixture of the MGDGs, including a glycerol moiety linked to a galactose unit and eicosapentaenoic acid combined with octadecatetraenoic acid or linolenic acid (IC_50_ = 65.70 µg/mL vs. 60.06 µg/mL, respectively). The NO inhibitory activity was also observed for several other seaweeds including *Ecklonia radiata, Hormosira banksia*, *Myriogloea sciurus*, *Phyllospora comosa*, *Solieria robusta*, and *Ulva* sp., with the NO inhibition being greatest in the nonpolar, lipid-rich DCM extracts (>76% activity for all species), followed by the intermediate polarity ethyl acetate extracts (>50% activity for all species except *H. banksia*), with the lowest activity observed in the polar butanol extracts [90].

Cytokines are potent signaling molecules that regulate immune responses and are secreted by the cells of innate and adaptive immunity in response to various stimuli [122]. Therefore, the assessment of the production of cytokines is another important parameter to consider in the evaluation of the anti-inflammatory activity of seaweeds. Pro-inflammatory cytokines contribute to the initiation and propagation of the immune response, whereas anti-inflammatory cytokines facilitate the attenuation of inflammation. Therefore, the balance between pro- and anti-inflammatory cytokines plays a significant role in the resolution of inflammation, and excessive production of pro-inflammatory cytokines may lead to chronic inflammation and has been linked with several inflammatory conditions [123,124].

The use of crude lipid extracts from seaweeds promoted down regulation of cytokines production in LPS stimulated THP-1 cells. Robertson et al. [98] observed that incubation of the LPS stimulated THP-1 cells incubation with *P. palmata* lipid extract reduced the secretion of IL-8 and IL-6. However, interestingly, *C. crispus* lipid extract significantly increased production of TNF-α. It should be noted that *C. crispus* is characterised by high content of ARA, which is recognised as a pro-inflammatory fatty acid. In addition, lipid extracts of *P. palmata*, *C. crispus*, and *P. dioica* promoted the downregulation of mRNA of Toll-like receptor 1 (TLR1) and Toll-like receptor 8 (TLR8), and TNF receptor associated factor 5 (TRAF5). In addition, *P. dioica* extract downregulated nine other pro-inflammatory genes including TLR4 and signal transducer and activator of transcription 3 (STAT3). However, lipid extracts of *P. palmata* and *P. dioica* upregulated NOS2 and PTGER1 gene expression. Another study reported that the lipid extract of *Macrocystis pyrifera* attenuated lipoteichoic acid (LTA) and LPS induced inflammatory response in THP-1 monocytes through suppression of MCP-1, IL-1β and IL-8, and MCP-1 secretion, respectively [107]. Moreover, *M. pyrifera* lipids reduced mRNA expression of MCP-1, IL-1β and IL-8 and myeloid differentiation primary response 88 (MYD88) and NFκB2/p100 protein levels and the phosphorylation of RelA/p65 in LTA-activated monocytes, which indicates the extract inhibits both canonical and non-canonical NFκB pathways [107].

The anti-inflammatory potential of seaweed lipids has also been evaluated in in vivo models (BALB/c mice). The 7-methoxy-9-methylhexadeca-4,8-dienoic acid (MMHDA) isolated from the brown seaweed *Ishige okamurae* demonstrated anti-inflammatory activity by reduction of phorbol 12-myristate 13-acetate (PMA) induced oedema and erythema in a mouse model [114]. The observed anti-inflammatory effect of MMHDA was linked to the inhibition of phospholipase A2 (PLA2) activity. The PLA2 catalysed hydrolysis of the membrane phospholipids is the primary pathway through which AA is liberated [125], which in turn is metabolized by COX-2 to prostaglandins that play a key role in the generation of the inflammatory response [116] Khan et al. [113] tested anti-inflammatory activity of stearidonic acid (SA), EPA and AA isolated from the brown seaweed *Undaria pinnatifida* in a BALB/c mice mode. SA and EPA significantly decreased PMA induced mouse ear inflammation by reducing oedema, erythema, and blood flow. Similar effects were observed for *U. pinnatifida* derived AA at low concentrations, but AA doses of more than 243 µg per ear demonstrated pro-inflammatory activity.

## 7. Conclusions

**Figure 2:**
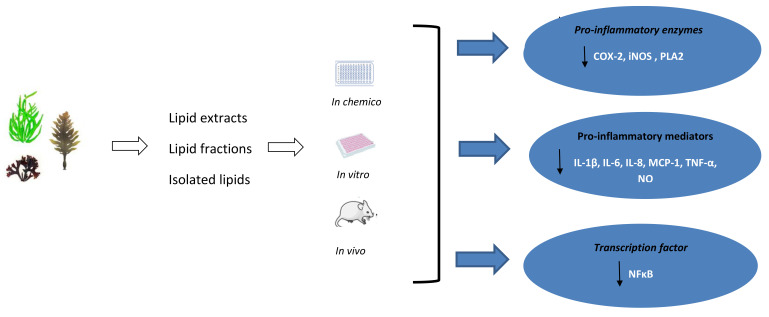
Schematic representation of the anti-inflammatory effects of seaweed lipids.

This review highlights that seaweeds are a rich, sustainable, and underexplored source of bioactive lipids with potent anti-inflammatory activity. The global burden of chronic, non-communicable diseases, including type 2 diabetes, hypertension, obesity, some types of cancer, and CVD put enormous pressure on public health services around the world. The evidence indicates that targeting inflammatory pathways could be a new strategy in the treatment and/or prevention of these chronic metabolic complications. Seaweeds are characterised by high nutritional value and their lipids can offer a number of bioactive compounds, including n-3 LC-PUFA, which may modulate the inflammatory response and reduce the risk of several NCDs. n-3 LC-PUFA are considered one of the most important anti-inflammatory components present in diets [126]. Thus, the inclusion of seaweed and seaweed lipids in a diet can provide preventive measures against several chronic inflammatory disorders. However, further investigations on the absorption and bioavailability of seaweed derived components are needed. In addition, any negative impact of the consumption of seaweed should be considered, especially excessive intake of iodine and heavy metals (mainly, arsenic), so there is a need to control the overall nutritional quality of seaweed and seaweed derived products [46]. To date, several studies reported anti-inflammatory properties of seaweed lipids that were associated with the suppression of iNOS and COX-2 expression and the reduction ofNF-κB/p100 and MYD88 protein levels that resulted in the downregulation of the production of several pro-inflammatory cytokines (TNF-α, IL-1β, IL-6, IL-8, MCP-1) and NO (Table 2, Figure 2). The most studied seaweed species were *Palmaria palmata*, *Chondrus crispus*, and *Porphyra doicia* from Rhodophyta phylum. It is worth noting that red seaweeds are charcaterised by the highest content of EPA and DHA when compared to other phyla (Table 1). The anti-inflammatory activity was observed for both crude lipid extracts, and isolated lipid fractions, including several GLs, and fatty acids (SA, AA, and EPA). Marine GLs are esterified with n-3 LC-PUFA, including EPA and DHA [127], unlike terrestrial plants, and it was suggested that PUFA side chains of galactolipids are essential for their anti-inflammatory activity [121]. Seaweed lipids are emerging as bioactive compounds with hidden immunomodulating potential.

As the majority of work focused on the evaluation of several pro-inflammatory mediators only there is limited evidence on the complex anti-inflammatory mechanisms of seaweed lipids (Table 2). The lack of comprehensive knowledge of the complexity of the inflammatory response hinders the understanding of links between seaweed bioactive lipids and their potential immunomodulating activity. Further investigations, including clinical studies with human participants, are needed to unravel the mechanisms underlying their actions against chronic inflammation and their potential therapeutic application.

Chronic inflammatory conditions contribute to more than 50% of all deaths globally [4], and seaweed derived lipids may be a potential new anti-inflammatory nutraceutical. Further research should focus on the identification, isolation and purification of seaweed lipid compounds and the links between their structure and mechanisms of anti-inflammatory activity is warranted. Seaweeds have been widely used in the food industry as a stabilizer, emulsifier, gelling and thickening agent to improve the shelf life, nutritional, textural, and organoleptic properties of different food products [39,127]. It was also reported that seaweed addition could increase n-3 LC-PUFA content and reduce the n-6:n-3 ratio in a variety of meat products [127]. However, more research is required to evaluate the potential application of seaweed lipids in health promoting foods and/or health supplements. In addition, innovative seaweed cultivation technologies to optimise lipid content and composition and to neutralise the effect of potentially harmful components need to be investigated. 

## Figures and Tables

**Table 2:** Anti-inflammatory activities of seaweed lipids.

Seaweed Species	Phylum	Type of Lipids/Lipid Extract	Model	Results	Reference
*Sargassum ilicifolium*	Ochrophyta	Crude lipid extract Extraction: methanol:chloroform 2:1	Murine macrophage RAW 264.7 cells NO inhibition	*S. ilicifocum* from Ujung Genteng Beach–Sukabumi region: NO inhibition ranged from 83.21% to 26.10% in pre-incubation model, and from 28.07 to 61.81% in co-incubation model *S. ilicifocum* from Awur Bay–Jepara region: NO inhibition ranged from 11.29 to 65.76% in pre-incubated cell culture model and 13.44–41.80% in co-incubation model No significant effect was observed for SHB treatment	[106]
*Porphyra dioica* *Palmaria palmate* *Chondrus crispus*	Rhodphyta	Crude lipid extract Extraction: methanol:chloroform 1:1	Human THP-1 monocytic cell line Production of pro-inflammatory cytokines Expression of genes linked to inflammatory signaling	Downregulation of *TLR4*, *STAT3*; upregulation of *PTGRE1*, Downregulated production of IL-6 and IL-8; upregulation of *NOS2* Upregulated production of TNF-α All species downregulated *TLR1*, *TLR8*, *TRAF5*	[98]
*Macrocystis pyrifera*	Ochrophyta	Crude lipid extract Extraction: methanol:chloroform 2:1	Human THP-1 monocytic cell line Production of pro-inflammatory cytokines NFκΒ pathway	Reduced production of MCP-1, IL-8 and IL-1β Reduced expression of mRNA of MCP-1, IL-8, and IL-1β Reduced levels of MyD88 and NFκΒ2/p100 protein	[107]
*Ulva rigida* *Codium tomentosum*	Chlorophyta	Crude lipids extract Extraction: methanol:chloroform 2:1	Cyclooxygenase Inhibition Assay	COX-2 inhibition of 87.9 ± 0.1% COX-2 inhibition of 82.3 ± 2.2%	[85]
*Gracilaria gracilis* *Palmaria palmate* *Porphyra dioica*	Rhodophyta	Crude lipids extract Extraction: methanol:chloroform 2:1	Cyclooxygenase Inhibition Assay	No inhibition of COX-2 COX-2 inhibition of 89.5 ± 0.9%, COX-2 inhibition of 83.6 ± 8.1%	[85]
*Fucus vesiculosus*	Ochrophyta	Crude lipids extract Extraction: methanol:chloroform 2:1	Cyclooxygenase Inhibition Assay	COX-2 inhibition of 34.6 ± 7.1%	[85]
*Grateloupia turuturu*	Rhodophyta	Crude lipids extract Extraction: methanol:chloroform 2:1	Cyclooxygenase Inhibition Assay	COX-2 inhibition of 50% at the concentration of lipid extract of 33 µg mL^−1^	[100]
*Palmaria palmata*	Rhodophyta	Crude lipids extract Extraction: methanol:chloroform 1:1 The extract partitioned with EtAOc and the EtAOc fraction, and next fractioned by silica gel column chromatography into three subfractions. 10 compounds were identified (2S)-1-O-eicosapentaenoyl-2-O-myristoyl-3-O-(6-sulfo-α-d-quinovopyranosyl)-glycerol (1), (2S)-1-O-eicosapentaenoyl-2-O-palmitoyl-3-O-(6-sulfo-α-d-quinovopyranosyl)-glycerol (2), 1-O-eicosapentaenoyl-2-O-trans-3-hexadecenoyl-3-phospho-(1′-glycerol)-glycerol (3), 1-O-eicosapentaenoyl-2-O-palmitoyl-3-phospho-(1′-glycerol)-glycerol (4), 1,2-Dieicosapentanoyl-glycero-3-phophocholine (5), (2S)-1,2-bis-O-eicosapentaenoyl-3-O-β-d-galactopyranosylglycerol (6), (2S)-1-O-eicosapentaenoyl-2-O-palmitoyl-3-O-β-d-galactopyranosylglycerol (7), (2S)-1,2-bis-O-eicosapentaenoyl-3-O-(β-d-galactopyranosyl-6-1α-d-galactopyranosyl)-glycerol (8), (2S)-1-O-eicosapentaenoyl-2-O-myristoyl-3-O-(β-d-galactopyranosyl-6-1α-d-galactopyranosyl)-glycerol (9) and (2S)-1-O-eicosapentaenoyl-2-O-palmitoyl-3-O-(β-d-galactopyranosyl-6-1α-d-galactopyranosyl)-glycerol (10)	Murine macrophage RAW 264.7 cell line NO production iNOS expression	The methanolic extract showed no effect on the NO production. The EtOAc fraction showed significant does-depended NO inhibition; at 100 μg/mL, the EtOAc fraction inhibited 89.4% of NO. All isolated lipids showed dose depended NO inhibitory activity and reduced iNOS expression.	[108]
*Ecklonia radiata* *Hormosira banksia* *Myriogloea sciurus* *Phyllospora comosa* *Solieria robusta* *Ulva* sp.	Ochrophyta Rhodophyta Chlorophyta	Crude lipid extract. Extraction: dichloromethane: methanol1:1, The crude extract was partitioned into DCM, EtOAc and BuOH.	Murine macrophage RAW 264.7 cell line NO production	Reduction in NO production across almost all extracts. The NO inhibitory activity was greatest in the nonpolar, lipid-rich DCM extracts (>76% activity for all species), followed by the intermediate polarity ethyl acetate (EtOAc) extracts (>50% activity for all species except *H. banksia*), with the lowest activity observed in the polar butanol (BuOH) extracts.	[90]
*Lobophora*	Ochrophyta	Crude lipids extract Extraction: methanol:chloroform 2:1 followed by the separation to polar and non-polar lipid fractions.	Murine macrophage RAW 264.7 cell line NO production	Non-polar lipid fraction displayed the highest NO inhibitory activity followed by crude lipid extract and polar lipid.	[109]
*Chondrus crispus*	Rhodophyta	Methanolic extract partitioned against EtOAc. The EtOAc fraction was subjected to solid phase extraction, followed by elution with hexane/EtOAc, CHCl_3_, and MeOH gradients to obtain three subfractions. 11 metabolites were identified: eicosapentaenoic (EPA, 1), arachidonic acids (AA, 2) and lutein (3), (2S)-1,2-Bis-O-eicosapentaenoyl-3-O-(β-D-galactopyranosylglycerol (4) (2S)-1-O-eicosapentaenoyl-2-O-arachidonoyl-3-O-β-D-galactopyranosylglycerol (5), (2S)-1-O-(6Z,9Z,12Z,15Z-octadecatetranoyl)-2-O-palmitoyl-3-O-β-D-galactopyranosylglycerol (6), (2S)-1-O-eicosapentaenoyl-2-O-palmitoyl-3-O-β-D-galactopyranosylglycerol (7), (2S)-1,2-bis-O-arachidonoyl-3-O-β-D-galactopyranosylglycerol (8), (2S)-1-O-arachidonoyl-2-O-palmitoyl-3-O-β-D-galactopyranosylglycerol (9), (2S)-1-O-eicosapentaenoyl-2-O-palmitoyl-3-O-(β-D-galactopyranosyl-6-1α-D-galactopyranosyl)-glycerol (10), and (2S)-1-O-arachidonoyl2-O-palmitoyl-3-O-(β-D-galactopyranosyl-6-1α-D-galactopyranosyl)-glycerol (11)	Murine macrophage RAW 264.7 cell line NO production	The methanolic extract showed does-dependent NO inhibition reduction by 15.6% of NO production at 100 μg mL^−1^ concentration. EtOAc fraction showed a fourfold increase in activity by inhibiting 64.6% NO production at 100 μg mL^−1^ concentration. Subfraction 1 (metabolites 1, 2, and 3) was relatively weaker in NO inhibitory activity, as compared to the remaining two polar fractions. Lutein and galactolipids showed the strongest NO inhibitory activity as compared to both free polyunsaturated fatty acids, EPA) and AA.	[110]
*Gracilaria* sp.	Rhodophyta	Crude lipid extract. Extraction: methanol:chloroform 2:1, followed by the fractionation of lipid extract to polar lipids.	Murine macrophage RAW 264.7 cell line NO production	Polar lipids showed a dose-dependent NO inhibition of 35% attained at the concentration of 100 µg/mL. The extract at concentrations lower than 50 μg/mL had no significant inhibitory effect on NO production.	[111]
*Fucus spiralis*	Ochrophyta	Methanolic extract was subjected to partitioning to give n-hexane, EtOAc and n-BuOH fractions. The EtOAc fraction was partitioned to: - monoacylglycerol featuring oleic acid (Compound (1)) - 1:1 mixture of two MGDGs containing eicosapentaenoic acid combined with octadecatetraenoic acid (Compound (2)) - 1:1 mixture of two MGDGs containing eicosapentaenoic acid combined with linolenic acid (Compound (3))	Murine macrophage RAW 264.7 cell line NO production	All isolated compounds showed dose dependent NO inhibitory activity. The fraction consisting of compounds (2) and (3), in a ratio of 1:1, was slightly more effective than compound 1.	[112]
*Undaria pinnatifida*	Ochrophyta	Acetonitrile extract fractioned by silica gel column chromatography. Three fatty acids were isolated: stearidonic acid (SA), eicosapentanoic acid (EPA), arachidonic acid (AA), at a flow rate of 2 mL/min.	Inflammatory bioassay: BALB/c mice; phorbol 12-myristate 13-acetate (PMA) and various concentrations of SA, EPA and AA were applied topically to the mouse ears. Ear oedema, erythema and blood flow were measured 10 h later.	SA: IC_50_ values of 160, 314, and 235 µg per ear for edema, erythema, and blood flow, respectively. EPA: IC_50_ values of 230, 462, and 236 µg per ear, edema, erythema and blood flow, respectively. AA: low concentrations showed anti-inflammatory activities, but doses of more than 243 µg per ear induced inflammatory symptoms.	[113]
*Ishige okamurae*	Ochrophyta	Methanolic extract fractioned by polarity. The moderately polar chloroform extract was further fractionated on a Sephadex LH-20 column. The most active fraction was further fractionated on a silica gel column with n-hexane. The late n-hexane eluant was applied to a reverse-phase HPLC. The most active peak was eluted at 25% acetonitrile and analysed with GS-MS. The isolated compound was 7-methoxy-9-methylhexadeca-4,8-dienoic (MMHDA)	Phospholipase A_2_ (PLA_2_) inhibition Inflammatory bioassay: BALB/c mice; phorbol 12-myristate 13-acetate (PMA) and MMHDA were applied topically to the mouse ears. Ear oedema and erythema were measured 10 h later.	Inhibition of PLA_2_, oedema and erythema. PLA_2_ IC_50_ and MIC 1.9 µg/mL and 4.0 µg/mL, respectively. Oedema IC_50_ and MIC 3.6 and 5.2 mg/mL, respectively. Erythema IC_50_ and MIC 4.6 mg/mL and 9.1 mg/mL, respectively.	[114]

## Data Availability

Not applicable.
